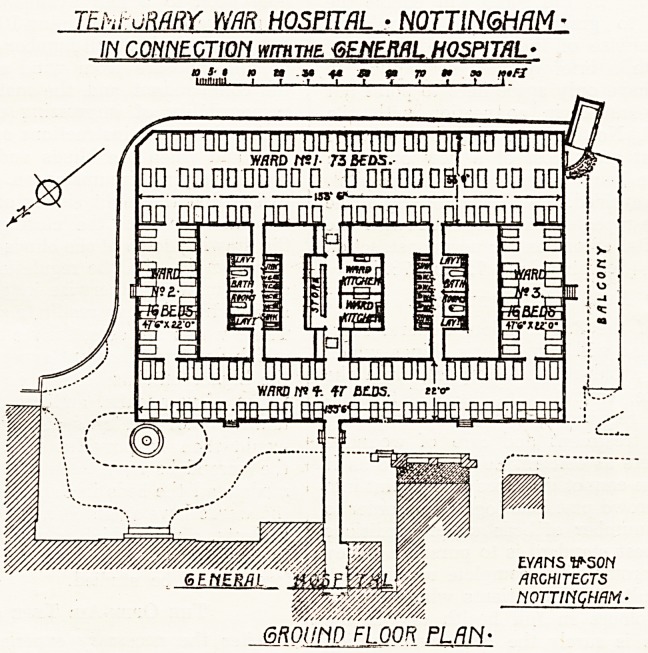# The Temporary War Hospital Attached to the General Hospital, Nottingham

**Published:** 1915-10-23

**Authors:** 


### THE TEMPORARY WAR HOSPITAL.

### Attached to the General Hospital, Nottingham.

We are indebted to the architects, Messrs. Evans and Son, for the following description of this hospital:

The Temporary War Hospital in connection with the General Hospital, Nottingham, has been recently completed, and is now partially occupied by wounded soldiers. The addition to the hospital provides accommodation for 152 beds, with the necessary kitchens, lavatories, stores, etc. Half the expense of erection and equipment has been defrayed by the War Office, and the remainder principally by the President of the hospital (Mr. W. G. Player). The only land available for the buildings in sufficiently close proximity to the hospital to permit of effective administration was the lawn on the south front, and hereupon a set of temporary wooden buildings has been erected. They comprise a rectangle of four wards, with seventy-three beds in the south ward, forty-seven in the north, and sixteen in each of the smaller wards.

In the inner court kitchen accommodation and sanitary conveniences, including a store for kit, and a series of bathrooms are so disposed as to be easily accessible to each of the wards. It should perhaps be explained that the kitchens are purely distributive apartments, the whole of the cooking being done in the main kitchen of the General Hospital itself. The buildings are bisected by a central corridor which communicates with the passage through the hospital proper to the entrance yard, so that the soldiers can be taken to the new quarters by a direct and level route. The wards and the accompanying erections are constructed of wood, the exteriors being coated with carbolineum, while the interiors are of three-ply boarding daintily durescoed in light green. The roofs are boarded and covered with Congo roofing, and the floors are of board. Electricity is the chief illuminant, and the heating is by steam, while the lavatory appliances and kitchens are supplied by water heated by steam through calorifiers.

### Ventilation and Cost.

In order to ensure thorough ventilation in all parts the buildings have been raised an average of two feet above the ground, the currents of air under the buildings flushing and sweetening the internal areas. That the wards are roomy and airy may be gathered from the fact that the average height is 13 ft., and that a floor space is allowed for each bed of from 66 ft. to 70 ft., while the cubic space per bed is approximately 800 ft. The south, or No. 1 ward, which is by far the largest, is 153 ft. long by 33 ft. wide. On the south-west front a verandah 20 ft. wide has been provided. The total cost of construction, including electricity, heating, and drainage, has not exceeded £20 per bed.

The hospital has been built to the plan of Messrs. Evans and Son, architects, of Nottingham; builders, Messrs. Gilbert and Hall, Nottingham; electrical engineers, Messrs. Thomas Danks and Co., Nottingham; heating engineers, Messrs. Ashwell and Nesbit, Leicester.

### Some Grave Defects.

In criticising the plan and considering the relation of the wards one to another, and their grouping round small central quadrangles, the area of which is much encroached upon by sanitary offices, we may at once say that we are disappointed. We had hoped that the disadvantages of such planning were so well known as to have secured its utter rejection for all time.

The long northern ward, while it suffers from its proximity to the permanent building, is dependent for its supply of sunlight on a number of windows looking into small land-locked court-yards. Even assuming that the site shown on the plan we publish was all the land available, we cannot help thinking that some better arrangement, and one more in accordance with the principles of hospital planning, could have been found.

When the fact that the floors are kept two feet above the ground with a free air space under is remembered, no knowledgeable critic can properly fail to condemn the crowding together of the wards on the site as shown on the plan. The resulting buildings cannot be accepted as ward blocks in which severe surgical cases should be treated. We do not know what method of classification of the patients has been adopted, but, whatever it may be, there can be no defence of a plan that forces a patient at any stage of his convalescence to traverse his own ward and a good portion of another before he can arrive at the corridor which leads to a sanitary convenience. It would be interesting to know who is responsible for the passing of this scheme and plan, and how Mr. Paul Waterhouse, M.A., F.R.I.B.A., regards both.

## Figures and Tables

**Figure f1:**